# LFG-500 Inhibits the Invasion of Cancer Cells via Down-Regulation of PI3K/AKT/NF-κB Signaling Pathway

**DOI:** 10.1371/journal.pone.0091332

**Published:** 2014-03-11

**Authors:** Chenglin Li, Fanni Li, Kai Zhao, Jing Yao, Yao Cheng, Li Zhao, Zhiyu Li, Na Lu, Qinglong Guo

**Affiliations:** State Key Laboratory of Natural Medicines, Jiangsu Key Laboratory of Carcinogenesis and Intervention, Key Laboratory of Drug Quality Control and Pharmacovigilance, Ministry of Education, China Pharmaceutical University, Nanjing, People's Republic of China; Xuzhou Medical college, China

## Abstract

Cancer cell invasion, one of the crucial events in local growth and metastatic spread of tumors, possess a broad spectrum of mechanisms, especially altered expression of matrix metalloproteinases. LFG-500 is a novel synthesized flavonoid with strong anti-cancer activity, whose exact molecular mechanism remains incompletely understood. This current study was designed to examine the effects of LFG-500 on tumor metastasis using *in vitro* and *in vivo* assays. LFG-500 could inhibit adhesion, migration and invasion of MDA-MB-231 human breast carcinoma cells. Meanwhile, it reduced the activities and expression of MMP-2 and MMP-9 via suppressing the transcriptional activation of NF-κB rather than AP-1 or STAT3. Moreover, LFG-500 repressed TNF-α induced cell invasion through inhibiting NF-κB and subsequent MMP-9 activity. Further elucidation of the mechanism revealed that PI3K/AKT but not MAPK signaling pathway was involved in the inhibitory effect of LFG-500 on NF-κB activation. LFG-500 could also suppress lung metastasis of B16F10 murine melanoma cells *in vivo*. Taken together, these results demonstrated that LFG-500 could block cancer cell invasion via down-regulation of PI3K/AKT/NF-κB signaling pathway, which provides new evidence for the anti-cancer activity of LFG-500.

## Introduction

Activating invasion and metastasis, the key hallmark of cancer [1], has been widely recognized as a primary cause of cancer-related mortality. Excessive basement membranes and extracellular matrix (ECM) degradation is crucial for tumor invasion and metastasis [2]. Matrix metalloproteinases (MMPs), a family of zinc-dependent endopeptidases, are associated with tumor cell invasion and metastasis [3], [4]. Tumor-secreted MMPs can hydrolyze ECM components in tissues surrounding the tumor, which facilitates the invasion of tumor cells through the basement membrane to distant organs and resulting in metastasis [5]. Among the MMPs, MMP-2 (gelatinase A, 72 kDa) and MMP-9 (gelatinase B, 92 kDa) play pivotal roles in ECM degradation [6]. Accordingly, they are abundantly expressed in various malignant tumors [7]. Therefore, MMPs and their regulatory pathways are considered as promising targets for anti-cancer drugs and chemotherapeutic agents [8].

It is generally demonstrated that phosphatidylinositol 3-kinase (PI3K)/AKT and mitogen- activated protein kinase (MAPK) signaling pathways regulate metastasis in a variety of cancer cells [9], [10]. The activation of downstream transcription factors including activator protein-1 (AP-1), STAT3, and nuclear factor-κB (NF-κB) are reported to stimulate the expression of MMPs at transcriptional level [11]. Especially, NF-κB, the extremely important transcription factor in cancer cells, has been implicated in many hallmarks of cancer development, including growth factor-independent proliferation, preventing apoptosis, unlimited replicative potential and tissue invasion and metastasis [12], [13]. NF-κB proteins comprise a family of structurally-related transcription factors, including p50 (NF-κB1), p65 (RelA), c-Rel, p52, and RelB. Among these factors, only p65, RelB, and c-Rel contain potent transactivation domains within sequences C-terminal to the Rel homology domain (RHD), which contains the DNA-binding and dimerization regions. Activated NF-κB is present in the nucleus where it binds to specific DNA sequences called response elements and regulates the transcription of target genes. While in the cytoplasm, NF-κB is kept in an inactive state, which complexes with the inhibitory IκB proteins. NF-κB can be activated through the classical IKK-dependent pathway leading to nuclear translocation of p50/p65 heterodimers [14].

**Figure 1:**
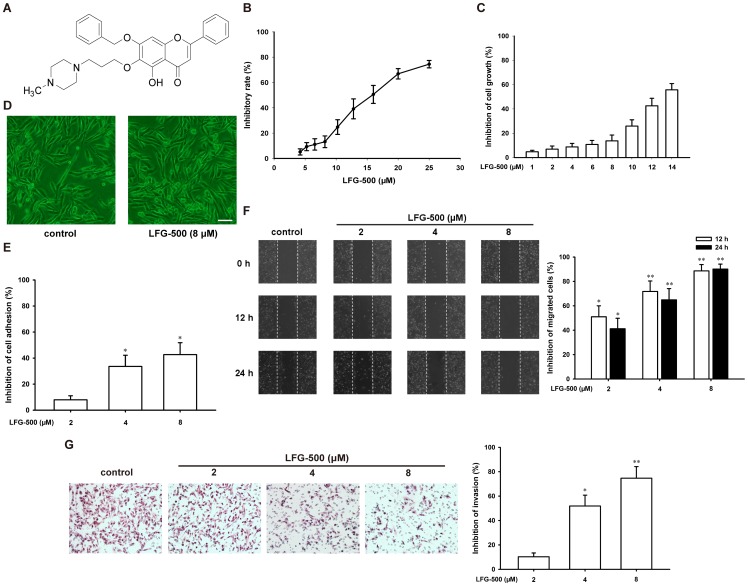
LFG-500 inhibits adhesion, migration, and invasion of MDA-MB-231 cells *in vitro*. (**A**) Chemical structure of LFG-500. (**B**) Inhibitory effect of LFG-500 on the growth of MDA-MB-231 cells for 24 h. MTT assay was employed. (**C**) Trypan blue dye exclusion assay of LFG-500 on the growth of MDA-MB-231 cells. Cells were treated with indicated concentrations of LFG-500 for 24 h. (**D**) LFG-500 (8 μM) has no influence on the normal size and shape of cells (×200, the scale bar represents 30 μm). (**E**) Inhibitory effect of LFG-500 on adhesion of MDA-MB-231 cells to fibronectin. Cell suspension (100 μl, 2×10^5^ cells/ml) was added to fibronectin pre-coated plates and incubated at 37°C for 1 h. Then culture media was carefully suctioned out. Each well was washed three times with PBS. MTT assay was adopted to determine the number of adherent cells. (**F**) LFG-500 inhibits cell migration. Cell monolayer was wounded by a 200 μL yellow pipette tip followed by treatment with various concentrations (2, 4, and 8 μM) of LFG-500 for 24 h. The number of the cells in the denuded zone was quantified under an inverted microscope. White lines indicate the wound edge. The migrated cells across the white lines were counted in six random fields from each treatment. Photographs of the wound of cells treated with LFG-500, ×100 (left). Quantification of the migrated cells (right). (**G**) LFG-500 inhibits cell invasion. Photographs of the invaded cell stained by hematoxylin and eosin, ×200 (left). Quantification of the invaded cells (right). *P<0.05 or **P<0.01 represents significant difference from the control group.

Flavonoids are a group of compounds rich in seeds, citrus fruits, olive oil, tea, and red wine. In recent years, the anti-tumor effects of flavonoids have been widely recognized and studied, especially their potent anti-metastasis activity [15], [16]. According to previous studies, flavonoids could inhibit invadopodia formation and MMP secretion [17] and prevent cell migration by up-regulating the expression of transgelin [18]. The multiple actions are attributable to their poly-phenolic structure. However, flavonoids have very low oral bioavailability due to its extensive first-pass metabolism, which would most likely happen at their hydroxyl groups. Therefore, based on the structure of flavonoids, LFG-500 (C_30_H_32_N_2_O_5_, **Fig. 1A**) was designed to improve oral bioavailability and prevent the metabolism of flavonoids at its hydroxyl groups by introducing a piperazine and a benzyl groups. These substitutions also gave LFG-500 better lipid solubility, making it more easily to enter into the intracellular space. Our previous study demonstrated that LFG-500 induced apoptosis through a reactive oxygen species (ROS)-mitochondrial-mediated pathway in HepG2 cells [19]. Since metastasis is extremely important in cancer progression, it is essential to investigate the effect of LFG-500 on cancer metastasis and the mechanism involved. The consequent findings can provide new evidence of the anti-cancer activity of LFG-500 as well as flavonoids.

## Materials and Methods

### Ethics Statement

This study was carried out in strict accordance with the recommendations in the Guide for the Care and Use of Laboratory Animals of the National Institutes of Health. The protocol was approved by the Committee on the Ethics of Animal Experiments of the China Pharmaceutical University. All surgery was performed under sodium pentobarbital anesthesia, and all efforts were made to minimize suffering.

### Materials

LFG-500 (99.1% purity), supplied by Professor Zhiyu Li (China Pharmaceutical University, China), was dissolved in dimethyl sulfoxide (DMSO) to a concentration of 10 mM as the primary stock solution. The solution was stored at −20°C, and diluted with medium before each experiment. The controls were treated with the same amount of DMSO (0.1%) as used in corresponding experiments. For *in vivo* study, LFG-500 solution was prepared by the School of Pharmacy in China Pharmaceutical University. Dacarbazine (DTTC, Nanjing pharmaceuticals company, China) was adopted as the positive control, which was dissolved in 0.9% NaCl before use. Fibronectin and matrigel were purchased from BD Biosciences (Bedford, MA, USA) [20]. Antibodies to MMP-9 (H-129) (sc-10737), MMP-2 (H-76) (sc-10736), c-Jun (D) (sc-44), c-Fos (4) (sc-52), NF-κB p65 (C-20) (sc-372), Lamin A (133A2) (sc-56137), IKKα/β (H-470) (sc-7607), IgG (H-270) (sc-66913), GFP (FL) (sc-8334), p-ERK1/2 (Thr 177/Thr 160)-R (sc23759-R), JNK (D-2) (sc-7345), p-JNK (G-7) (sc-6254), p38 (H-147) (sc-7149), p-p38 (Thr 180) (sc-101758), AKT1/2/3 (H-136) (sc-8312), β-actin (sc-130301), and protein A-agarose (sc-2001) were obtained from Santa Cruz Biotechnology (Santa Cruz, CA, USA). Antibodies to STAT3 (T721) (BS-1336), p-STAT3 (S727) (BS-4180), p-STAT3 (Y705) (BS-4181), ERK1/2 (L352) (BS1112), PI3K p85α/γ (Y463) (BS-3006), and p-AKT (S246) (BS-4286) were purchased from Bioworld (St. Louis Park.nneapoli, MN, USA). Antibodies to p-IKKα/β (Ser176/180) (16A6) (#2697), IκBα (44D4) (#4812), and p-IκBα (Ser32) (14D4) (#2859) were from Cell Signaling Technology, Inc. (Beverly, MA, U.S.A). MTT, trypan blue, gelatin, paraformaldehyde, formaldehyde, glycine, Triton X-100, DAPI, Tris, NaCl, Hepes, KOH, KCl, EDTA, NP-40, PMSF, NaF, SDS, DTT, and NaHCO_3_ were purchased from Sigma-Aldrich (St. Louis, MO, USA).

### Animals

Male C57BL/6 mice (6–8 weeks old) weighting 20–25 g were obtained from the Shanghai Laboratory Animal Center (Shanghai, China). The animals were maintained in a temperature and humidity controlled environment with a 12:12-h light/dark cycle. Feed and water were available *ad libitum*. All animal experimental and surgical procedures were strictly performed in accordance with the Guide for the Care and Use of Laboratory Animals.

### Cell culture

MDA-MB-231 (a human breast carcinoma cell line) and B16F10 (a highly metastatic murine melanoma cell line) were purchased from the Cell Bank of the Shanghai Institute of Cell Biology (Shanghai, China). MDA-MB-231 cells and B16F10 cells were cultured in Leibovitz's L15 and DMEM medium (Gibco, Invitrogen, Carlsbad, CA, USA), respectively, both of which contain 10% fetal bovine serum (Gibco, Invitrogen, Carlsbad, CA, USA), 100 U/mL penicillin (Beyotime, Nantong, China), and 100 μg/mL streptomycin (Beyotime, Nantong, China). The cells were maintained in a humidified atmosphere of 95% air/5% CO_2_ at 37°C.

### Colorimetric MTT assay

Cells (10^4^/well) were seeded on Falcon 96-well plates (BD Biosciences, Bedford, MA, USA) for 24 h and then exposed to different concentrations of LFG-500. After incubation for 24 h, 20 μL of 5 mg/mL MTT was added to the medium, and the cells were incubated at 37°C for another 4 h. Then the culture medium was discarded and 100 μL DMSO was added to each well to dissolve the precipitate. The absorbance (A) was measured at 570 nm using an Automated Microplated Reader ELx800 (BioTek Instruments, Inc. Winooski, VT, USA). Cell growth inhibitory rate (I%) was calculated by the following equation:
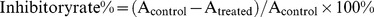
where A_treated_ and A_control_ were the average absorbance of three parallel experiments from treated and control groups, respectively. IC_50_ was taken as the concentration that caused 50% inhibition of cell growth and was calculated by the Logit method.

### Trypan blue dye exclusion assay

After exposed to LFG-500 at indicated concentrations for 24 h, cells were harvested and mixed with 0.4% trypan blue. Then, both unstained (viable) and stained (nonviable) cells were counted separately with a cell counting chamber (Qiujing, Shanghai, China) under the microscope (CX21; Olympus, Tokyo, Japan).

### Cell morphological assessment

Cells were seeded in 6-well cell culture plates (Corning, New York, NY, USA) and treated with LFG-500 (8 μM) for 24 h. At the end of incubation, cell morphology was monitored using an inverted light microscope (IX51, Olympus Corp., Tokyo, Japan).

### Cell adhesion assay

Cell adhesion assay was performed as described [20] with modifications. 96-well plates were coated with fibronectin (5 μg/mL) at 4°C overnight and then blocked in BSA (1%) for 1 h. Serum-starved cells were exposed to LFG-500 (2, 4, or 8 μM) for 24 h before seeding. Target cells were harvested and suspended in serum-free medium. The cells (2×10^5^/mL) were seeded to fibronectin-coated plates and then incubated at 37°C for 1 h. Non-adherent cells were removed by gentle washing with PBS. Then, colorimetric MTT assay was employed to analyze the adhesion ability of cells.

### Wound healing assay

Cells were seeded in 6-well plate and allowed to grow to 80% confluence. Subsequently, cell monolayer was scratched with a pipette tip (Axygen, Union City, CA, USA) to create a narrow wound-like gap. Shortly after wounding, the cells were washed with PBS twice and incubated with LFG-500 (2, 4, or 8 μM). The plates were photographed at 0, 12, and 24 h using an inverted light microscope. The number of migrated cells was quantified by manual counting and six randomly chosen fields were analyzed for each well [21].

### Cell invasion assay

Transwell chamber system (10 mm diameter, 8 μm pore size with polycarbonate membrane, Corning Costar, Cambridge, MA) coated with matrigel was employed to examine the invasive ability of cancer cells *in vitro* as described previously [22]. Briefly, transwell chambers were initially coated with matrigel (40 μg/100 μL/chamber) at 37°C for 1 h. The cells treated with or without LFG-500 (2, 4, or 8 μM, 24 h) were suspended in Leibovitz's L15 medium (5×10^5^ cells/ml) and seeded in the upper compartment, while the medium containing 10% fetal bovine serum was added in the lower compartment. After incubation in a humidified atmosphere of 95% air/5% CO_2_ at 37°C for 24 h, the non-invaded cells on the upper side of the membrane were removed with a cotton swab. The invaded cells on the bottom surface were fixed with 100% methanol and stained with hematoxylin and eosin (Nanjing Sunshine Biotechnology Ltd., Nanjing, China). The invaded cells were quantified by manual counting and six randomly chosen fields were analyzed for each group.

### Gelatin zymography assay

Cells were treated with or without LFG-500 (2, 4, or 8 μM) in serum-free medium for 24 h, and then the supernatants were collected for the samples. Gelatin zymography assay was performed according to previous method [16]. After treatment, enzyme-digested regions were observed as white bands against blue background. The zones of enzymatic activity were seen as negatively stained bands.

### Real-time PCR analysis

Cells were treated with LFG-500 (2, 4, or 8 μM) for 12 h, and then the levels of MMP-9 and MMP-2 mRNA were determined. The primer sequences synthesized by Sangon Biotech Co., Ltd. (Shanghai, China) were listed as follows: MMP-9: 5′-GCA GAG GAA TAC CTG TAC CGC-3′ (forward) and 5′-AGG TTT GGA ATC TGC CCA GGT-3′ (reverse); MMP-2: 5′-CAG GCT CTT CTC CTT TCA CAA C-3′ (forward) and 5′-AAG CCA CGG CTT GGT TTT CCT C-3′ (reverse); β-actin: 5′-CTG TCC CTG TAT GCC TCT G-3′ (forward) and 5′-ATG TCA CGC ACG ATT TCC-3′ (reverse). Reactions were conducted as described [23]. Samples were run on the ABI 7500 Real-Time PCR system (ABI, Foster City, CA, USA) as follows: 30 s at 95°C, followed by 40 cycles of 95°C for 5 s and 60°C for 34 s. Each reaction was done in triplicate. The threshold values (Cs) for each mRNA were subtracted from that of β-actin mRNA, averaged, and converted from log-linear to linear terms. Data were analyzed with the SDS 2.1 program.

### Western blot analysis

Cells were treated with various concentrations of LFG-500 (2, 4, or 8 μM) for 24 h, then collected and lysed. Western blotting analysis was conducted according to previous methods [24]. Detection was performed with the Odyssey Infrared Imaging System (LI-COR Inc., Lincoln, NE, USA). All blots were stripped and reprobed with polyclonal anti-β-actin to verify equal protein loading.

### Preparation of cytosolic and nuclear extracts

Collected cells were lysed with buffer A (10 mM Hepes-KOH (pH 7.9), 10 mM KCl, 0.1 mM EDTA, 0.5% NP-40, 1 mM DTT, and 0.5 mM PMSF) on ice for 15 min to allow cells to swell, and then centrifuged at 14 000×*g* at 4°C for 15 min. The supernatants were saved as the cytosolic fractions. The nuclear pellets were incubated with high-salt buffer (20 mM Hepes, 0.5 M KCl, 1 mM EDTA, 1 mM DTT, and 1 mM PMSF, pH 7.9) on ice for 30 min, and then centrifuged at 12 000 rpm at 4°C for 15 min to obtain nuclear fractions.

### Immunofluorescence detection

Cells were seeded on glass coverslips and incubated with or without 8 μM LFG-500 for 24 h. Following steps were performed as described [24] with modifications. Cells were fixed with 4% paraformaldehyde in PBS at 1-h intervals, permeabilized with 0.5% Triton X-100, and blocked with 2% BSA for 30 min. Incubation with primary antibody against NF-κB p65 (diluted 1∶50) was done overnight at 4°C. After washing, the cells were sequentially exposed to FITC-conjugated secondary antibodies (1∶1000, Invitrogen, Carlsbad, CA, USA, L42001) and DAPI. Images were observed and captured with a confocal laser scanning microscope (FV1000; Olympus, Tokyo, Japan).

### Electrophoretic mobility shift assay (EMSA)

Cells were treated with 2, 4, and 8 μM LFG-500 for 24 h. The DNA binding activities of NF-κB in nuclear extracts was assessed by EMSA [25] using the EMSA kit (Beyotime, Nantong, China) with biotin-labeled double-stranded NF-κB oligonucleotides (Beyotime, Nantong, China). The sequences of the oligonucleotides adopted were as follows: 5′-AGT TGA GGG GAC TTT CCC AGG C-3′ and 3′-TCA ACT CCC CTG AAA GGG TCC G-5′. Briefly, nuclear extracts (6 μg/sample) were incubated with the oligonucleotides in reaction buffers for 30 min. Protein DNA complexes were separated on a 6% nondenaturing acrylamide gel, transferred to positively charged nylon membranes, and cross-linked in a Stratagene cross-linker. Band shifts were detected by chemiluminescence method with Chemidoc XRS + System (Bio-Rad, Hercules, CA, USA).

### Chromatin immunoprecipitation (ChIP) assay

Cells were incubated with LFG-500 (2, 4, or 8 μM) for 24 h, and then ChIP assay was performed as described [26] with some modifications. In brief, the cells were cross-linked with formaldehyde, quenched with glycine, sonicated on ice, and centrifuged at 4°C. Aliquots of lysates containing 200 μg of protein were used for each immunoprecipitation reaction with anti-NF-KB p65 or pre-immune IgG. The mixtures were rotated at 4°C overnight and then incubated with protein A-agarose for 6 h. Finally, the captured immune complexes were eluted with elution buffer (1% SDS and 0.1 M NaHCO_3_) at 37°C for 30 min. Protein-DNA crosslinks were reversed at 65°C for 4 h in a high salt buffer (0.2 M NaCl, 50 mM Tris, pH 6.5, 10 mM EDTA, and 0.2 mg/ml Proteinase K). Extracted and dissolved immunoprecipitated DNA was quantified by real-time PCR analysis with primers encompassing the NF-κB binding sites. The primers for MMP-9 promoter quantification were 5′-CAG TGG AAT TCC CCA GCC TTG CCT-3′ and 5′-CCA CAC TCC AGG CTC TGT CCT C-3′.

### Cell transfection and NF-κB-dependent reporter gene expression assay

The effect of LFG-500 on NF-κB-dependent reporter gene transcription was analyzed by luciferase reporter gene assays [27]. Cells (5×10^5^ cells/well) were seeded on 6-well plates. Then the pNF-κB-luc (Beyotime, Nantong, China) containing four NF-κB binding motifs (GGGAATTTCC) was transiently transfected into the cells using lipofectamine 2000 (Invitrogen Inc., Grand Island, NY, USA), according to the manufacture's instruction. The pCDNA3.2 plasmid was added to make the total amount of DNA equal (4 μg/well in a 6-well plate) with GFP served as normalization control. Following LFG-500 (2, 4, or 8 μM) treatment for 24 h, luciferase assays were performed with the Luciferase Reporter Gene Assay kit (Promega, Madison, WI, USA) using Luminoskan ascent (Thermo, Waltham, MA, USA).

### In vivo tumor metastasis assay

B16F10 melanoma cells were collected to an appropriate concentration in PBS and injected via tail vein into syngeneic C57BL/6 mice. The mice were equally randomized into five groups (10 mice/group): 0.9% normal saline control group, DTTC 100 mg/kg/2 days positive control group, LFG-500 12.5 mg/kg/day group, LFG-500 25 mg/kg/day group, and LFG-500 50 mg/kg/day group. After inoculation for 24 h, the mice were intraperitoneally injected with test compounds for 21 days. Then the mice were weighed and sacrificed. White blood cell (WBC) was analyzed by the Sysmex K-4500 hematology analyzer (Sysmex Inc., Kobe, Japan). The lungs were removed, and washed with PBS. The number of surface tumor nodules was counted under a dissecting microscope [20].

### Statistical analysis

All data in the different experimental groups are expressed as the mean ± S.E.M. The data shown were obtained from at least three independent experiments. Differences between the groups were assessed by one-way ANOVA and Dunnett's post hoc test. Significant differences were represented as *P<0.05 or **P<0.01.

## Results

### LFG-500 inhibits adhesion, migration, and invasion of MDA-MB-231 cells in vitro

MTT assay showed that cell growth decreased systematically with increasing concentrations of LFG-500 (**Fig. 1B**). The IC_50_ value of LFG-500 on MDA-MB-231 cells for 24 h was 15.67±0.82 μM. However, LFG-500 (up to 8 μM) had no obvious influence on the growth of MDA-MB-231 cells (**Fig. 1B**), which was confirmed by trypan blue dye exclusion assay (Fig. 1C). Cell morphology examination also showed that LFG-500 (8 μM) did not affect the normal size and shape of cells (**Fig. 1D**). Therefore, 8 μM of LFG-500 was determined as the highest concentration for the subsequent experiments.

Tumor metastasis occurs through a complex series of events. Tumor cells exhibit a variety of properties, including altered adhesiveness, increased motility and invasive capacity, to complete the metastatic process [28]. Therefore, tumor cell adhesion to ECM and basement membrane is considered as a fundamental step for cancer metastasis. We examined the influence of LGF-500 on the adhesive ability of MDA-MB-231 cells to the substrates pre-coated with fibronectin, one important component of ECM. LFG-500 treatment (4 and 8 μM) suppressed MDA-MB-231 cells adhesion to fibronectin by 33.6±8.6% (n = 3, *P*<0.05) and 42.7±9.1% (n = 3, *P*<0.05), respectively (**Fig. 1E**). Moreover, the effects of LFG-500 on cell migration and invasion are detected. As shown in **Fig. 1F**, the migrated cells across the wounded space were decreased by 41.2±8.4% (n = 3, *P*<0.05), 64.9±9.2% (n = 3, *P*<0.01), and 89.1±4.2% (n = 3, *P*<0.01), respectively, when the cells were treated with 2, 4, and 8 μM LFG-500 for 24 h. In addition, LFG-500 (4 and 8 μM) reduced the invasion ability of MDA-MB-231 cells by 51.9±9.3% (n = 3, *P*<0.05) and 74.7±10.2% (n = 3, *P*<0.01), respectively (**Fig. 1G**).

### LFG-500 suppresses the activity and expression of MMP-2/9 in MDA-MB-231 cells

**Figure 2:**
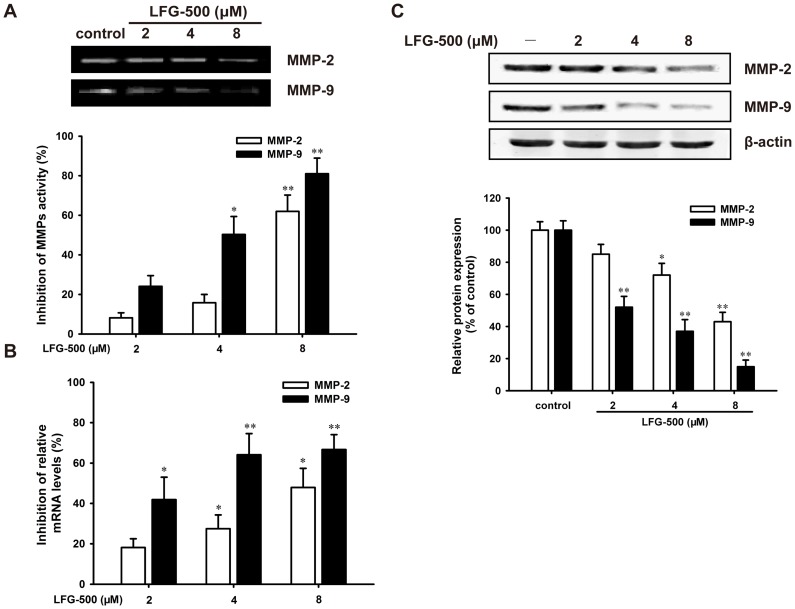
LFG-500 suppresses the activity and expression of MMP-2/9 in MDA-MB-231 cells. (**A**) LFG-500 inhibits the activity of MMP-2/9 in MDA-MB-231 cells. Cells were treated with various concentrations (2, 4, and 8 μM) of LFG-500 for 24 h. The conditioned media were collected, and then MMP-2/9 activity was determined by gelatin zymography assay. (**B**) LFG-500 decreases the levels of MMP-2/9 mRNA. Cells were incubated with indicated concentrations of LFG-500 for 12 h. The mRNA levels were detected by real-time PCR analysis. (**C**) LFG-500 suppresses the protein expression of MMP-2/9. MDA-MB-231 cell lysates were subjected to immunoblotting with antibodies against MMP-2 (1∶400) and MMP-9 (1∶400). *P<0.05 or **P<0.01 represents significant difference from the control group.

MMP-2/9, secreted and activated by cancer cells, hydrolyze the basement membrane and ECM, which facilitates the invasion of malignant cells and results in metastasis [29]. To explore the possible anti-metastasis mechanism of LFG-500, the gelatinolytic activity of MMP-2/9 secreted from MDA-MB-231 cells was detected following LFG-500 treatment. As shown in **Fig. 2A**, LFG-500 (8 μM) obviously suppressed the activity of MMP-2 and MMP-9, with inhibition rates of 62±8.2% (n = 3, *P*<0.01) and 81±7.9% (n = 3, *P*<0.01), respectively. Moreover, the inhibitory effect on the activity of MMP-9 was more significantly.

In order to further understand the down-regulatory effects of LFG-500 on MMP-2 and MMP-9, real-time PCR analysis was performed. As shown in **Fig. 2B**, the inhibition of gene expression was observed obviously, with the level of MMP-2 mRNA reduced by 47.5±9.5% (n = 3, *P*<0.05) and MMP-9 mRNA decreased by 68.1±7.1% (n = 3, *P*<0.01), respectively, following the treatment of 8 μM LFG-500 for 12 h. The LFG-500-mediated changes in the levels of MMP-2 and MMP-9 mRNA were consistent well with their protein expression, as evidenced by Western blot analysis (**Fig. 2C**). These results indicated that LFG-500 might regulate MMP-2/9 at the transcriptional level. More importantly, the inhibitory effects on MMP-9 were more noticeable.

### LFG-500 represses cell invasion through inhibiting the transcriptional activation of NF-κB and subsequent MMP-9 activity

**Figure 3:**
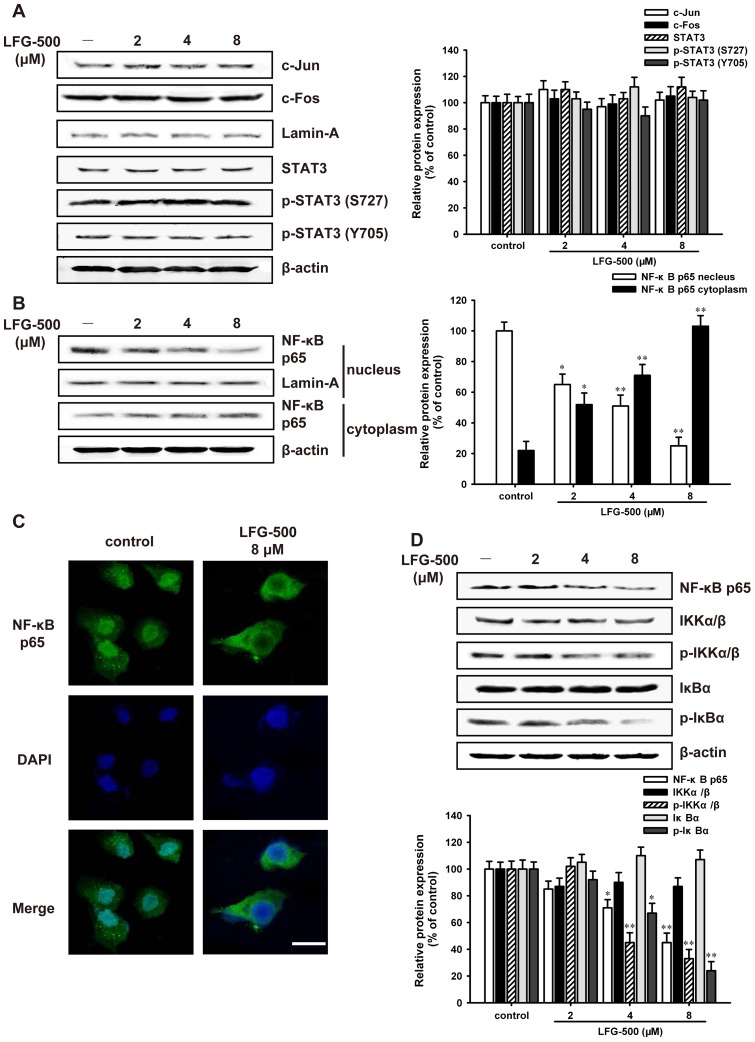
LFG-500 inhibits the nuclear translocation of NF-κB rather than AP-1 or STAT3 in MDA-MB-231 cells. (**A**) LFG-500 has no significant effects on AP-1 or STAT3. Cells were treated with various concentrations (2, 4, and 8 μM) of LFG-500 for 24 h. The nuclear levels of the proteins were detected by Western blot assay using specific antibodies. Lamin-A was used as nuclear loading control. (**B**) LFG-500 inhibits the nuclear translocation of NF-κB. Cytosolic and nuclear extracts in the cells were prepared according to the “Materials and methods”. The nuclear and cytoplasmic levels of NF-κB p65 were determined by Western blot assay. (**C**) The inhibitory effect of LFG-500 on the nuclear translocation of NF-κB. MDA-MB-231 cells were immunostained with anti-NF-κB p65 (1∶50) and DAPI (×400, the scale bar represents 30 μm). (**D**) The effects of LFG-500 on phosphorylation levels of IKKα/β and IκBα. Cells were treated with various concentrations (2, 4, and 8 μM) of LFG-500 for 24 h, and the expression of target proteins was detected by Western blotting using specific antibodies where β-actin was used as loading control. *P<0.05 or **P<0.01 represents significant difference from the control group.

It is generally reported that transcriptional regulation by activating transcription factors including AP-1 [30], STAT3 [31], or NF-κB [32] occurs during the modulation of MMP-9 gene expression. To further clarify the mechanism underlying LFG-500 suppressing MDA-MB-231 cell invasion, we investigated the effects of LFG-500 on these transcription factors. The data in **Fig. 3A** demonstrated that LFG-500 had no significant effects on the nuclear levels of c-Jun or c-Fos (components of AP-1). Besides, there was no noticeable change in the phosphorylation of STAT3 when given the same treatment (**Fig. 3A**). However, LFG-500 inhibited the nuclear translocation of NF-κB p65, with a decreased nuclear level but an increased cytoplasmic level (**Fig. 3B**), which was confirmed by immunofluorescence assay (**Fig. 3C**). Moreover, the total amount of NF-κB p65 was decreased after LFG-500 treatment (**Fig. 3D**). For the reason that NF-κB activation results from rapid phosphorylation, ubiquitination, and ultimately proteolytic degradation of IκB [33], as well as IKK is required for phosphorylation of IκB [34], the phosphorylation levels of IKKα/β and IκBα were detected. LFG-500 (4 µM and 8 µM) efficiently inhibited the phosphorylation of IKKα/β and IκBα, whereas the total steady-state levels remained unchanged (**Fig. 3D**). These results indicated that NF-κB rather than AP-1 or STAT3 might be involved in the anti-invasive effect induced by LFG-500.

**Figure 4:**
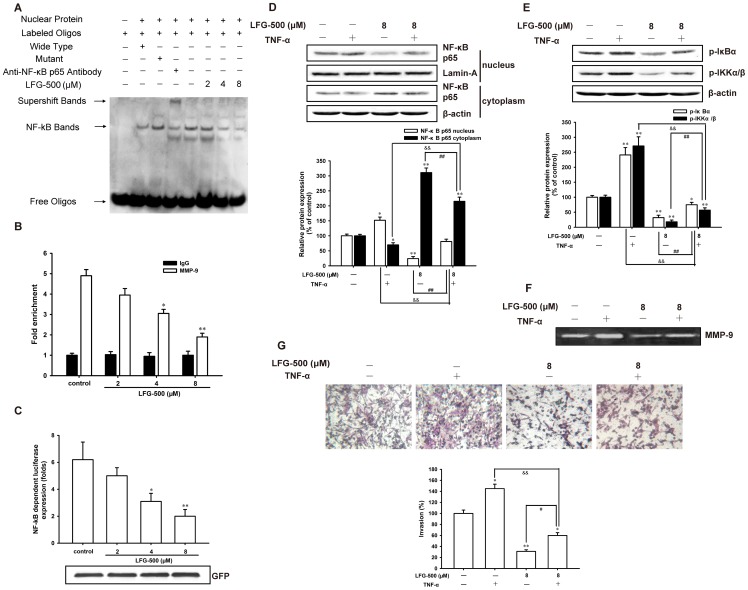
LFG-500 represses cell invasion through inhibiting the transcriptional activation of NF-κB and subsequent MMP-9 activity. (**A**) LFG-500 suppresses the DNA-binding activity of NF-κB. The binding activity of nuclear extracts to oligonucleotides was detected by EMSA. A labeled probe containing the area of NF-κB binding site was utilized, and the bolt was representative of three experiments. (**B**) The binding activity of NF-κB to MMP-9 promoter following LFG-500 treatment was determined by ChIP assay. The relative enrichment is shown. (**C**) LFG-500 inhibits the activity of NF-κB-dependent reporter. The pNF-κB-luc plasmid containing four NF-κB binding motifs (GGGAATTTCC) was transiently transfected into the cells. After treatment with LFG-500 (2, 4, or 8 μM) for 24 h, luciferase assays were performed where GFP served as normalization control. (**D**) LFG-500 (8 µM) inhibits the nuclear translocation of NF-κB induced by TNF-α, as well as TNF-α-activated phosphorylation of IκBα and IKKα/β (**E**). Cells were stimulated with TNF-α (20 ng/mL) for 1 h. The expression of target proteins was detected by Western blotting analysis. Densitometric analysis of the proteins studied (down). (**F**) LFG-500 suppresses the activity of MMP-9 induced by TNF-α. Gelatin zymography assay was used. (**G**) LFG-500 inhibits cell invasion induced by TNF-α. Photographs of the invaded cell stained by hematoxylin and eosin, ×200 (up). Quantification of the invaded cells (down). *P<0.05 or **P<0.01 represents significant difference from the control group. ^&&^ *P*<0.01 represents significant difference between TNF-α group and TNF-α + LFG-500 group. *^#^P*<0.05 or *^##^P*<0.01 represents significant difference between LFG-500 group and TNF-α + LFG-500 group.

Subsequently, the DNA-binding activity of translocated NF-κB was further evaluated. *In vitro*, the nuclear extracts were incubated with DNA probes specific for NF-κB, and their binding was assessed by mobility shift. As shown in **Fig. 4A**, LFG-500 remarkably blocked the DNA-binding activity of NF-κB. To further confirm this finding, ChIP assay was performed to test the binding activity of NF-κB to the promoter of MMP-9, which is the NF-κB target gene. Results showed that the binding activity was significantly inhibited following LFG-500 treatment (**Fig. 4B**). Because DNA-binding alone is not always correlate with NF-κB-dependent gene transcription [35], the effects of LFG-500 on NF-κB-dependent reporter activity was also analyzed. MDA-MB-231 cells were co-transfected with GFP and pNF-κB-luc plasmids. LFG-500 obviously suppressed luciferase activity, with about a 3-fold decrease following 8 μM of treatment (**Fig. 4C**). These results indicated that LFG-500 could inhibit the transcriptional activation of NF-κB.

TNF-α is one of the most potent activators of NF-κB and its activation mechanism is relatively well established [36]. Accordingly, we examined the effect of LFG-500 on TNF-α-induced NF-κB activation in MDA-MB-231 cells. Treatment with LFG-500 (8 µM) could substantially inhibit the nuclear translocation of NF-κB induced by TNF-α (**Fig. 4D**), as well as the TNF-α-activated phosphorylation of IκBα and IKKα/β (**Fig. 4E**), which meant that LFG-500 could weaken the activation of NF-κB induced by TNF-α. More importantly, LFG-500 repressed the activity of MMP-9 (**Fig. 4F**) and subsequent cell invasion (**Fig. 4G**) induced by TNF-α. These results further indicated that LFG-500 suppressed cell invasion by inhibiting NF-κB activation and subsequent MMP-9 activity.

### PI3K/AKT signaling pathway is involved in the inhibitory effect of LFG-500 on NF-κB activation

**Figure 5:**
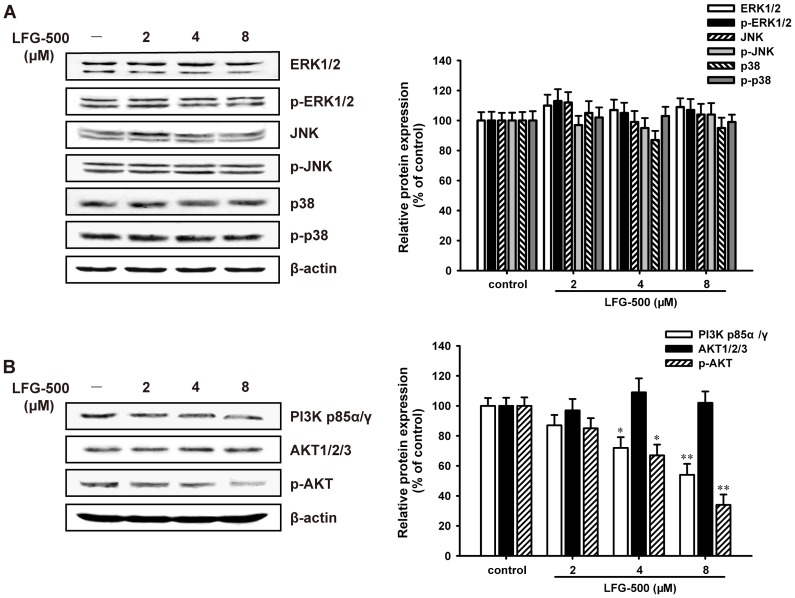
PI3K/AKT signaling pathway is involved in the inhibitory effect of LFG-500 on NF-κB activation. (**A**) LFG-500 has no influence on MAPK signaling pathway. MDA-MB-231 cells were treated with various concentrations (2, 4, and 8 μM) of LFG-500 for 24 h. Total expression and phosphorylation of ERK, JNK, and p38 were analyzed by Western blotting. β-actin was used as a loading control. (**B**) LFG-500 inhibits the expression of PI3K p85α/γ and p-AKT. The expression of target proteins was determined by Western blotting. Densitometric analysis of the proteins studied (right). *P<0.05 or **P<0.01 represents significant difference from the control group.

Data in **Fig. 3D** indicated that LFG-500 suppressed NF-κB activation at least partly through inhibiting the phosphorylation of IKKα/β and IκBα. It is reported that the MAPK and PI3K/AKT pathways can regulate fundamental cellular processes by induction of IKK dependent NF-κB activation [37], [38]. Therefore, the effects of LFG-500 on MAPK and PI3K/AKT signaling pathways were determined. As shown in **Fig. 5A**, LFG-500 had little effect on MAPK family including JNK, ERK and p38. These results confirmed the finding that LFG-500 exerted no significant effect on AP-1 (**Fig. 3A**), because AP-1 can be activated by the MAPK signaling pathway [39]. However, LFG-500 significantly reduced the levels of PI3K and p-AKT, while the total steady-state level of AKT remained unchanged (**Fig. 5B**). These data indicated that LFG-500 could inhibit the activation of NF-κB through PI3K/AKT rather than MAPK signaling pathway.

### LFG-500 suppresses lung metastases of tumor in vivo

**Figure 6:**
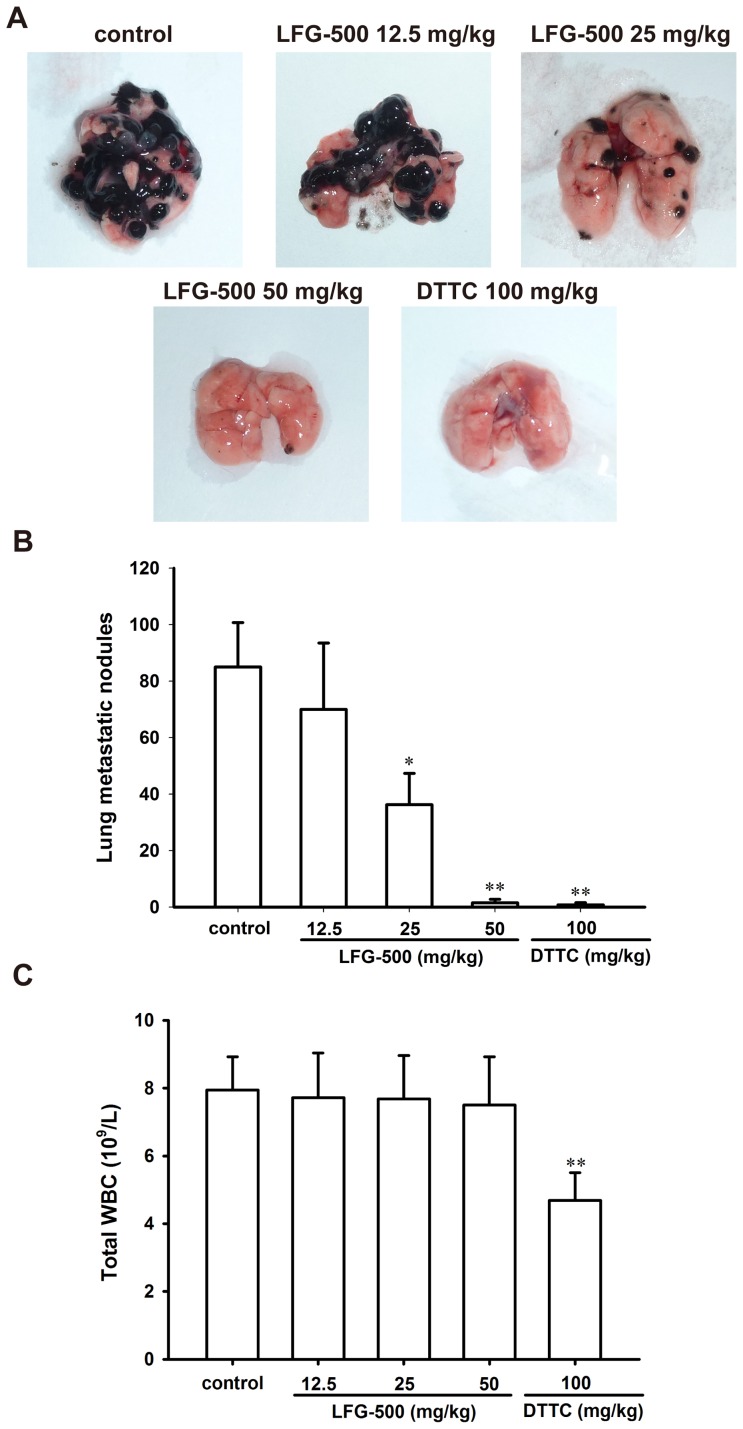
LFG-500 suppresses lung metastases of mouse melanoma B16F10 cells *in vivo*. (**A**) The representative images for the lung metastatic nodules of different treatment groups. (**B**) Quantification of the number of lung metastatic nodules. (**C**) The WBC count of C57BL/6 mice in different groups. *P<0.05 or **P<0.01 represents significant difference from the control group.

To further confirm the anti-metastatic effect of LFG-500 *in vivo*, an artificial lung metastatic model was employed. DTTC, the first choice for melanoma treatment in clinical setting, was used as the positive control. As shown in the representative images, LFG-500 (25 mg/kg and 50 mg/kg) significantly suppressed lung metastasis in mice compared with the control group (**Fig. 6A**). The number of lung metastatic nodules in the control group was 85±15.7, while only 36.2±11.1 and 1.5±1.2 nodules were observed in the 25 mg/kg group and 50 mg/kg group (**Fig. 6B**), respectively. Moreover, LFG-500 had no obvious influence on white blood cell (WBC) count (**Fig. 6C**). In contrast, DTTC could inhibit lung metastases to a significant extent, but it did suppress WBC count.

## Discussion

Breast cancer is the most common cancer around the world, and also the second leading cause of death owing to cancer in women in the United States [40]. In most cases, death results from the metastasis and proliferation of cancer cells at secondary sites. Tumor metastasis is a complex and multistage process, and tumor cells are required to express a variety of properties including altered adhesiveness, increased motility and invasive capacity to complete the metastatic process [41]. Moreover, degradation of stromal ECM is a crucial step in tumor invasion and metastasis. Interruption of this step may be a strategy for prevention and treatment of breast cancer metastasis. Anti-metastasis drugs involved in these mechanisms have been extensively investigated [42]. Flavonoids, as one important group of natural products derived from flavone, are widely investigated for their variety of anti-cancer activities. LFG-500 is a newly synthesized flavonoid with a piperazine and a benzyl group (**Fig. 1A**). In the present study, we demonstrated that LFG-500 could inhibit adhesion, migration, and invasion of MDA-MB-231 human breast carcinoma cells *in vitro*. Then we investigated whether LFG-500 plays a role in tumor metastasis *in vivo*, by establishing a spontaneous and experimental B16F10 melanoma metastasis mode which is convenient and intuitive. Results showed that LFG-500 significantly suppressed the lung metastasis *in vivo*. This effect could be explained by suppressing PI3K/AKT/NF-κB signaling pathway, which consequently inhibited the activity of MMP-9, and in turn leading to the reduced invasiveness of the cancer cells.

Tumor progression is associated with the activity of MMPs at multiple stages, such as tumor establishment, growth, neovascularization, and metastasis [43]. An enhanced expression of MMPs, particularly the gelatinase (MMP-2 and MMP-9), is associated with high metastasis potential in several types of human carcinomas including breast cancer [44]. To further explore the mechanism of LFG-500-induced inhibitory effect on migration and invasion, the activity and expression of MMP-2/9 in MDA-MB-231 cells were detected. LFG-500 notably downregulated the activity of MMP-2 and MMP-9 (**Fig. 2A**) as wells as the protein expression (**Fig. 2C**) through suppressing the transcriptional activity of MMP-2 and MMP-9 genes in MDA-MB-231 cells (**Fig. 2B**). More importantly, the inhibitory effect on MMP-9 was more noticeable. Therefore, it is worthwhile to elucidate the mechanism underlying LFG-500 regulating the activity and expression of MMP-9 at transcriptional level.

The transcription of MMP-9 gene is regulated by upstream regulatory elements, such as AP-1, STAT3, and NF-κB binding sites [45]–[47]. Actually, one or more of these binding sites have been implicated in mediating the effects of a diverse set of agents. Consequently, suppression of the activity of AP-1, STAT3, or NF-κB binding to respective regulatory elements potentially inhibits tumor invasion [48]. LFG-500 had no significant effects on the transcriptional activation of AP-1 or STAT3 (**Fig. 3A**). However, LFG-500 significantly inhibited the nuclear translocation of NF-κB (**Fig. 3B, 3C**), DNA-binding activity (**Fig. 4A, 4B**) as well as NF-κB-dependent reporter activity (**Fig. 4C**), which suggested that LFG-500 could repress the transcriptional activation of NF-κB rather than AP-1 or STAT3 in MDA-MB-231 cells. Moreover, LFG-500 inhibited the cell invasion induced by TNF-α (**Fig. 4F, 4G**), one of the most potent activators of NF-κB. These data further indicated that LFG-500 suppressed cell invasion by inhibiting NF-κB activation and subsequent the activity of MMP-9. Meanwhile, NF-κB, the most important transcription factor in cancer cells, regulates the expression of genes responsible for transformation, tumor promotion, tumor invasion, angiogenesis, and metastasis as well as suppressing apoptosis, which participates in the progress of carcinogenesis [49], [50]. The inhibitory effect of LFG-500 on NF-κB activity might result in other multiple responses in cancer cells.

It is accepted that the activation of MAPK or PI3K/AKT signaling pathway is important for regulating NF-κB activation and subsequent the activity of MMP-9 in response to different stimulators in several cell lines [51], [52]. To investigate the inhibitory mechanism of LFG-500 on NF-κB activation, the two key signaling pathways were identified. PI3K/AKT but not MAPK signaling pathway was involved in the inhibition of LFG-500 on NF-κB activation (**Fig. 5**), which indicated that LFG-500 could suppress PI3K/AKT/NF-κB signaling pathway in MDA-MB-231 cells. Moreover, a major mechanism essential for human cancer progression is the PI3K/AKT signaling pathway [9]. The capability of this pathway to induce deregulated proliferation and survival of human cancer cells may depend not only on the activation of genes that stimulate cellular proliferation, migration, and metastasis, but also on the inhibition of those genes that suppress proliferation and/or induce apoptosis [53]. The inhibitory effect on PI3K/AKT signaling pathway might also regulate other transcription factors for suppressing cell invasion. Besides, previous work demonstrated that the apoptosis of HepG2 cells induced by LFG-500 was related to activated JNK and p38 MAPK pathways [19]. The various effects on MAPK signaling pathway might due to different concentrations of LFG-500 trigger multiple processes necessary for carcinogenesis. The underlying mechanism needs further study.

Nevertheless, further research is required for better understanding the mechanism of LFG-500 to inhibit tumor invasion in our future study. For example, tissue inhibitors of matrix metalloproteinases (TIMPs), which are naturally occurring MMP inhibitors, can form 1∶1 stoichiometric complexes with MMPs to suppress the activities of their pro-enzymes, representing important physiological regulators of matrix turnover [54]. It is possible that TIMP-1 is involved in the inhibitory activity of MMP-9 when exposure to LFG-500. In addition, the epithelial-mesenchymal transition (EMT) plays a pivotal role in tumor progression and metastasis, which endows epithelial cells with enhanced metastatic and invasive potential [55]. Such a process requires the involvement of activated NF-κB [56], and we have determined that LFG-500 can block the activation of NF-κB (**Fig. 4**). Besides, a recent study has demonstrated that natural flavonoid can suppress bladder cancer metastasis via inhibiting β-catenin/ZEB1 signaling and subsequent EMT [57]. These data indicate that LFG-500 might also suppress cancer metastasis by regulating EMT. However, it is just a possible mechanism involved, and we will focus on this point in the near further. Moreover, LFG-500 has been found to strongly block tumor cell invasion using a B16F10 melanoma metastasis model (Fig. 6). The ability of this agent to inhibit MDA-MB-231 cells in an orthotopic transplantation tumor model needs further investigation.

**Figure 7:**
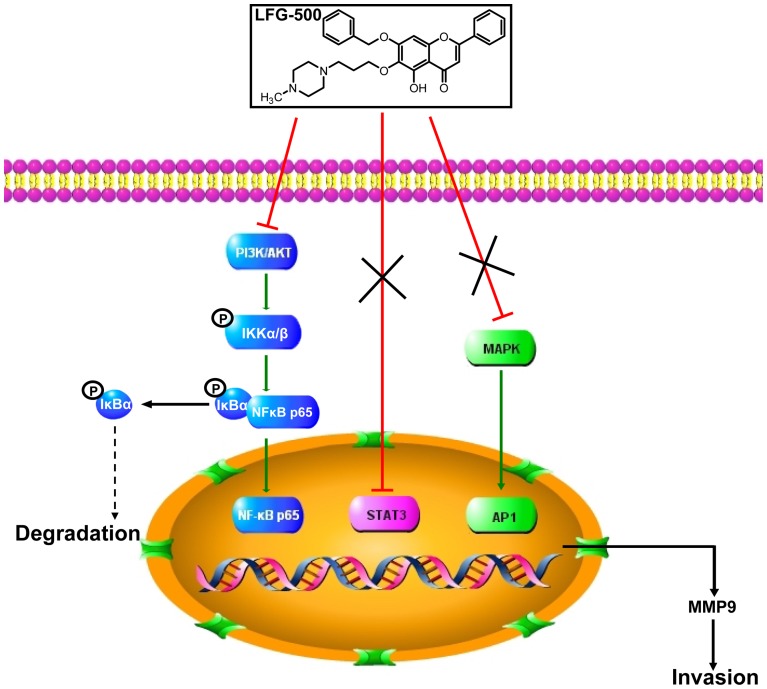
A possible mechanism underlying the inhibitory effect of LFG-500 on the invasion of cancer cells.

In conclusion, our studies demonstrate that LFG-500 significantly inhibits the invasive ability, activity and expression of MMP-9 in MDA-MB-231 cells by suppressing PI3K/AKT/NF-κB signaling pathway (**Fig. 7**). Therefore, these findings provide new evidence for better understanding the anti-metastatic activity of LFG-500, which can facilitate further investigation on its potential of anti-cancer therapy.
